# Comparative study of each surgical step in radical prostatectomy under 3D and 2D laparoscopy

**DOI:** 10.3389/fsurg.2024.1347583

**Published:** 2024-01-31

**Authors:** Pengcheng Zhang, Yuhan Pei, Yunlai Zhi, Ninghong Song, Fanghu Sun

**Affiliations:** ^1^ Department of Urology, Lianyungang Clinical College of Nanjing Medical University, The First People’s Hospital of Lianyungang, Lianyungang, China; ^2^ Department of Urology, The First Affiliated Hospital of Nanjing Medical University, Nanjing, China

**Keywords:** radical prostatectomy, prostate cancer, 3D laparoscopy, 2D laparoscopy, surgical steps

## Abstract

**Objective:**

Comparing the specific advantages and surgical outcomes of each step in radical prostatectomy under 3D vs. 2D laparoscopy.

**Methods:**

From October 2019 to January 2023, our urology department treated 63 cases of prostate cancer, using an odd-even arrangement method to divide into two groups. This is a non-randomized prospective study, with 33 odd-numbered cases in the 3D group and 30 even-numbered cases in the 2D group. The surgery was divided into four steps: (1) establishing an extraperitoneal pneumoperitoneum (2) pelvic lymph node dissection (3)excising the prostate (4)bladder-urethral anastomosis, comparing the two groups in terms of surgical time, blood loss, and relevant postoperative indicators for each step.

**Results:**

All 63 surgeries were successfully completed without any conversions. Comparing 3D and 2D laparoscopy groups, there were statistically significant differences in total surgery time (123.5 ± 15.3 min vs. 145.6 ± 17.2 min, *P* < 0.05), total blood loss (198.3 ± 18.4 ml vs. 243.1 ± 20.1 ml, *P < *0.05), prostate excision time (55.1 ± 8.4 min vs. 67.2 ± 9.3 min, *P < *0.05) and blood loss (101.6 ± 12.2 ml vs. 123.8 ± 14.1 ml, *P < *0.05), bladder-urethral anastomosis time (30.5 ± 4.3 min vs. 37.6 ± 5.1 min, *P < *0.05) and blood loss (62.7 ± 9.7 ml vs. 82.5 ± 8.2 ml, *P < *0.05). There were no statistical differences in the time and blood loss during the establishment of extraperitoneal pneumoperitoneum and the cleaning of pelvic lymph nodes (*P > *0.05). In terms of urinary incontinence rates, the 3D laparoscopy group was lower than the 2D group, and in terms of preserving erectile function, the 3D group was higher than the 2D group, with significant statistical differences (*P < *0.05). There were no statistically significant differences between the two groups in terms of postoperative drainage days, hospitalization days, hospitalization costs, time of catheter removaland positive margin rates (*P > *0.05).

**Conclusion:**

Compared to traditional 2D laparoscopy, 3D laparoscopy can shorten the operation time and reduce bleeding in the steps of prostate excision and bladder-urethral anastomosis, but there was no significant difference in peri-operative outcomes.

## Introduction

Prostate cancer is a common male disease in urology, ranking first in the incidence of male malignant tumors in 105 countries worldwide (1–3). With the development and popularization of laparoscopy, its significant superiority has been demonstrated. Laparoscopy has been successfully applied to radical prostatectomy; with accumulated experience and technical improvements, it significantly reduces intraoperative bleeding and complications compared to open surgery, gaining widespread recognition (4). However, laparoscopy is performed under a two-dimensional view, and the images are flat, lacking a three-dimensional sense, which makes it difficult for surgeons to judge distance and depth, affecting the precision of the surgery.

3D laparoscopy is a new device that has been gradually developed in recent years, overcoming the aforementioned limitations of two-dimensional (2D) laparoscopy, providing a three-dimensional visual effect similar to open surgery, thereby reducing the difficulty of surgery and enhancing precision (5, 6). Clinical application has found that in radical prostatectomy, 3D laparoscopy can provide an enlarged high-definition three-dimensional view, clearly displaying the tissue anatomy and the direction of vascular and neural spaces, greatly reducing the difficulty of anatomical dissection of the prostate, and maximally protecting key prostate tissues and their functions. With the widespread use of 3D laparoscopy, some disadvantages have been identified: (1) prolonged operations or rapid movement of the camera may cause discomfort such as dizziness in the surgeon; (2) the camera cannot rotate, unlike 2D laparoscopy, which can obtain different views by rotating the camera angle; (3) it is prone to interference with other instruments when operating in areas like the pelvis. Additionally, some 3D devices have heavy cameras, which can be physically demanding for assistants who need to hold the laparoscope for extended periods.

Previous comparative studies on 3D and 2D laparoscopic radical prostatectomy have mostly focused on the total surgery time and blood loss, noting that 3D laparoscopy reduces both, but without specifying which steps are affected. Laparoscopic radical prostatectomy is complex and time-consuming, with varying degrees of complexity and duration for each step. 3D laparoscopy offers advantages in reducing surgery time and blood loss, but also has the aforementioned shortcomings, and its advantages may vary at each step of the surgery. In response to this, from October 2019 to January 2023, we subdivided the surgery into four steps for clinical research: (1) establishing extraperitoneal pneumoperitoneum, (2) pelvic lymph node dissection, (3) excising the prostate, and (4) bladder-urethral anastomosis. We completed 33 cases of radical prostatectomy under 3D laparoscopy and 30 cases using traditional 2D laparoscopy for prostate malignancy during the same period. The specific advantages and disadvantages of each step and the surgical outcomes of both methods were compared, and the report is as follows.

## Materials and methods

### Instrument and equipment

3D Laparoscopic Surgical System Viking Systems, USA.

2D Laparoscopic Surgical System Richard Wolf Endoscopy, Germany.

### Clinical information

From October 2019 to January 2023, 63 prostate cancer patients were admitted according to inclusion criteria and divided into two groups using an odd-even arrangement method: the odd-numbered as the 3D group and the even-numbered as the 2D group. In the 3D group (33 cases), the ages ranged from 57 to 75 years, with an average of 68.3 years. BMI ranged from 19.8 to 28.6 kg/m², averaging 22.9 kg/m². Prostate volumes were 51.8–67.3 ml, averaging 58.3 ml. Gleason scores ranged from 4.8 to 8.1, averaging 7.2. Prostate-specific antigen (PSA) levels were 10.4–18.3 ng/ml, averaging 13.59 ng/ml. In the 2D group (30 cases), the ages ranged from 58 to 77 years, with an average of 69.7 years. BMI ranged from 20.5 to 29.1 kg/m², averaging 23.5 kg/m². Prostate volumes were 53.1–69.7 ml, averaging 61.3 ml. Gleason scores ranged from 4.9 to 8.2, averaging 7.3. PSA levels were 10.1–17.9 ng/ml, averaging 12.45 ng/ml. After statistical analysis, there were no significant differences between the two groups in terms of age, BMI, prostate volume, and Gleason score, indicating that the cases were comparable (*P* > 0.05) (detailed in Table 1).

**Table 1:** Demographics and preoperative characteristics.

Parameter	3D group	2D group	*t*/*χ^2^*	*p*-value
Age (years)	68.3 ± 5.6	69.7 ± 7.3	−1.978	0.174
BMI (kg/m2)	22.9 ± 1.8	23.5 ± 2.1	0.216	0.963
Prostate volume (ml)	58.3 ± 13.3	61.3 ± 16.8	0.972	0.398
Gleason score (points)	7.2 ± 0.9	7.3 ± 1.1	0.917	0.412
PSA level (ng/ml)	13.59 ± 2.9	12.45 ± 2.3	1.319	0.293
Clinical T stage (*n*)			0.617	0.129
T1a/b	3	2		
T1c	12	11		
T2a/b	10	9		
T2c	5	4		
T3a	3	4		

Inclusion criteria for both groups: (1) Transrectal ultrasound-guided 13-core prostate biopsy, with pathology confirming prostate adenocarcinoma, Gleason score 4–9; (2) Pelvic MRI and isotope whole body bone imaging to exclude surrounding organ and bone metastases of the prostate; (3) No other serious comorbidities, including coronary artery atherosclerotic heart disease, cerebral infarction, severe hypertension, and diabetes. (4) No other surgical contraindications. Exclusion criteria: (1) Incomplete clinical data; (2) Severe cardiopulmonary dysfunction, coexisting autoimmune diseases; 3) Patients with a severe tendency to bleed, poor coagulation function; (4) Presence of pelvic or other tumors.

### Operation procedure

**Figure 1:**
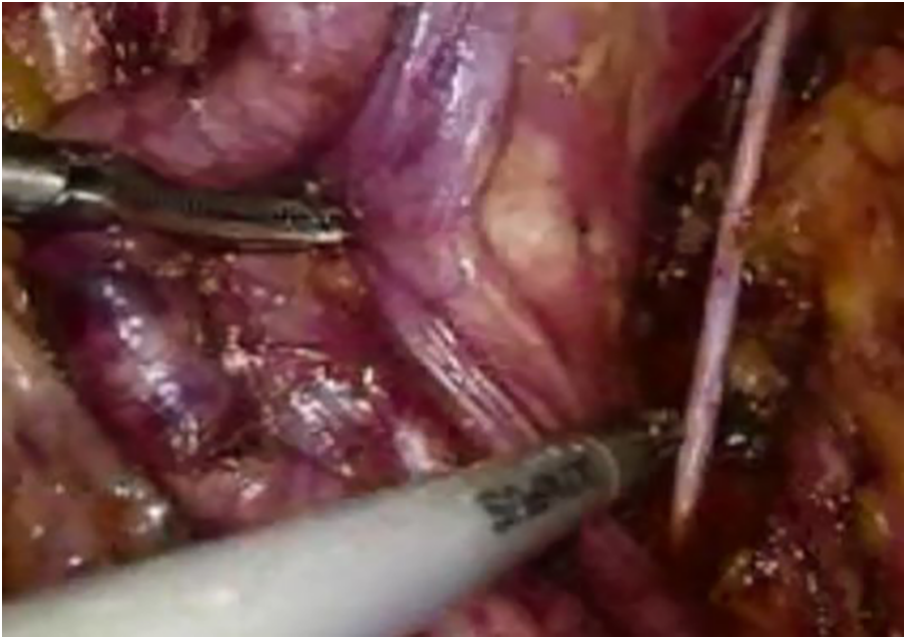
Pelvic lymph node dissection.

The procedure is divided into the following four steps: Step One (Figure 1): Establishing the extraperitoneal pneumoperitoneum. A 2.0 cm vertical incision is made below the navel, the skin is incised, subcutaneous tissue bluntly separated, the anterior sheath of the rectus abdominis opened, the extraperitoneal space expanded with the fingers, and a homemade water balloon is inserted with 800–1,000 ml of water to further expand the extraperitoneal space.

**Figure 2:**
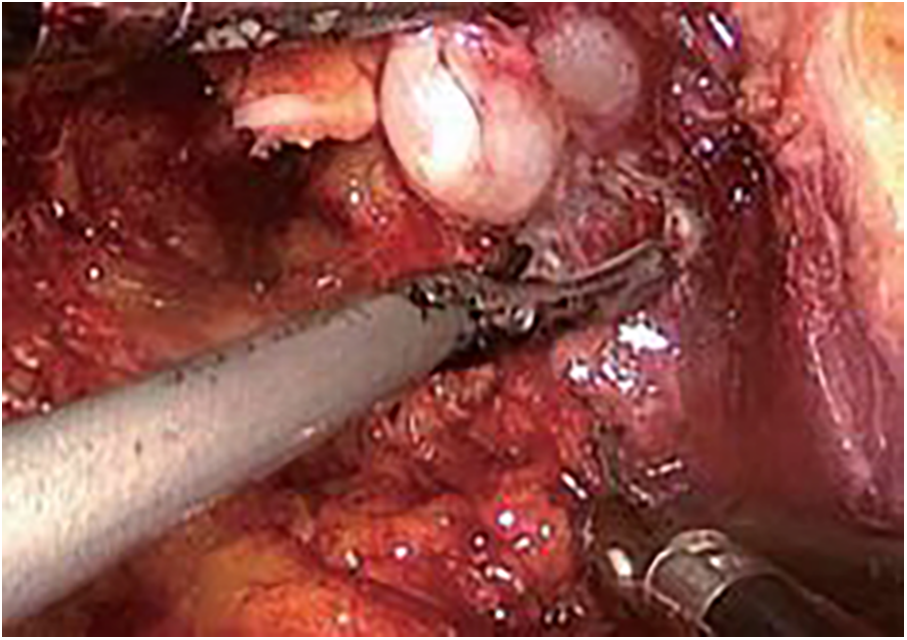
Seminal vesicle mobilization.

Step Two (Figure 2): Pelvic lymph node dissection. Starting from the bifurcation of the iliac vessels, the external iliac artery, vein, and obturator nerve are dissected out, and the surrounding fat and relevant lymphatic tissue are completely excised to achieve “skeletonization,” followed by pathological biopsy.

**Figure 3:**
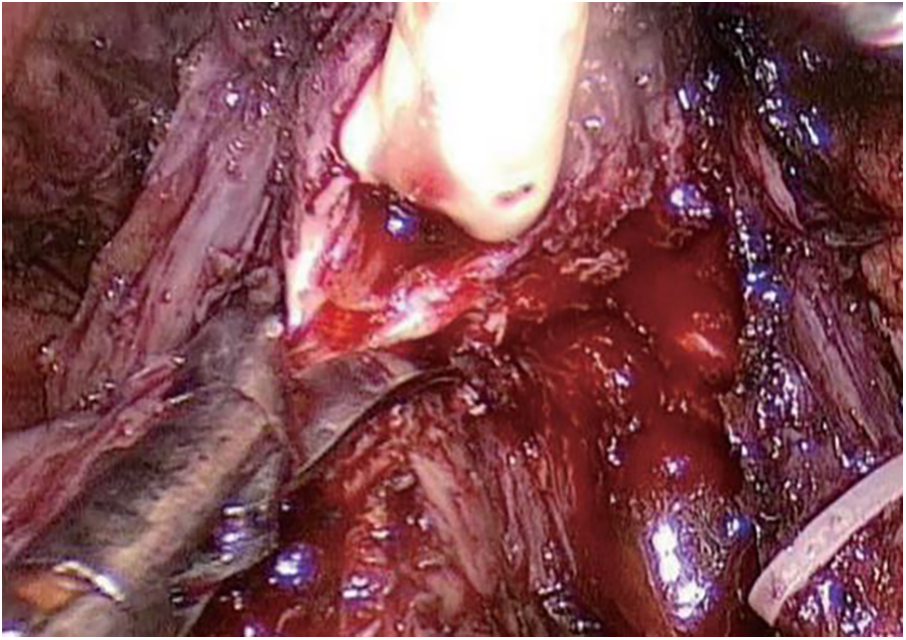
Transection of the urethra.

Step Three (Figure 3): Prostate excision. Incise the anterior wall of the bladder, cut the connection between the prostate and the bladder, free and pull out the seminal vesicles from the posterior aspect. Mobilize the posterior wall of the prostate to the apex, and ligate the deep dorsal vein complex at the severed and ligated base. Below the apex of the prostate, transect the urethra, and completely excise the prostate.

**Figure 4:**
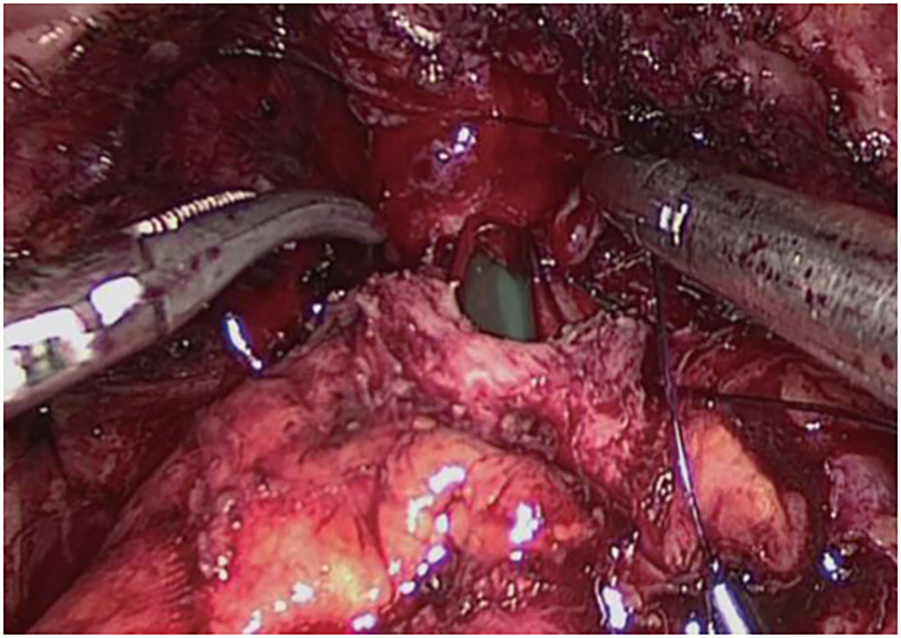
Bladder-urethral anastomosis.

Step Four (Figure 4): Bladder-urethral anastomosis. Starting at the 3 o'clock position of the bladder neck, the needle is inserted from the outside to the inside of the bladder neck, and from the inside to the outside at the corresponding position of the urethra. Perform 6–8 continuous sutures, complete the circumferential anastomosis, pull both ends of the thread, tighten and knot the anastomosis, inject 100 ml of water into the bladder to check for leaks, and complete the bladder-urethral anastomosis.

### Data and statistics

In this study, a total of 2 lead surgeons performed the surgeries, both possessing 20 years of surgical experience, and the surgical procedures have been standardized. Statistical analysis of both groups was conducted on surgery time and blood loss for each surgical step, postoperative drainage days, hospitalization days, hospitalization costs, postoperative urinary incontinence incidence, positive surgical margin rates, and preservation of erectile function. Complete urinary control is defined as no longer using pads or using only one preventive pad. The Assessment timepoint of continence control was after removal of the catheter. The incidence rate of urinary incontinence refers to the occurrence rate after the removal of the catheter. Postoperative erectile function is assessed using the International Index of Erectile Function-5 (IIEF-5), which includes 5 questions, each rated on a 6-point scale (0–5 points). The total score ranges from 0 to 25, with a score ≥22 indicating normal erectile function and <22 indicating erectile dysfunction (ED). The Assessment timepoint of IIEF-5 is 1 month after surgery. Blood loss was calculated separately for each step during the surgery. Calculation of blood loss after transecting the bladder neck and urine outflow: Corrected based on preoperative hemoglobin content of the patient, i.e., actual blood loss = volume of fluid collected from the wound × hemoglobin concentration of the collected fluid/preoperative hemoglobin concentration. Data were analyzed using SPSS 22.0 software. Quantitative data were presented as (x¯ ± s), comparisons between the two groups were made using independent sample *t*-tests, qualitative data were analyzed using chi-square tests, and *P < *0.05 was considered statistically significant.

## Results

### Intraoperative indicators

Both groups successfully completed the surgeries without any conversions. The 3D group had significantly less operative time and blood loss during the prostate excision and bladder-urethral anastomosis stages compared to the 2D group, with a statistically significant difference (*P < *0.05). There was no statistically significant difference in operative time and blood loss during the establishment of extraperitoneal pneumoperitoneum and pelvic lymph node dissection stages between the two groups (*P > *0.05). Among the cases in both groups, the bladder neck was preserved in 40 cases and not preserved in 23 cases (detailed in Table 2).

**Table 2:** Intraoperative indicators.

	3D group	2D group	*t*	*p*-value
Total surgery time (min)	123.5 ± 15.3	145.6 ± 17.2	−8.791	0.012
1. Time to establish extraperitoneal pneumoperitoneum (min)	11.4 ± 3.3	12.5 ± 2.9	1.817	0.428
2. Time for pelvic lymph node dissection (min)	26.5 ± 4.7	28.3 ± 5.6	2.089	0.341
3. Prostate excision time (min)	55.1 ± 8.4	67.2 ± 9.3	6.891	0.026
4. Bladder-urethral anastomosis time (min)	30.5 ± 4.3	37.6 ± 5.1	7.019	0.022
Total blood loss (ml)	198.3 ± 18.4	243.1 ± 20.1	9.128	0.009
1. Blood loss in establishing extraperitoneal pneumoperitoneum (ml)	1.5 ± 0.2	1.6 ± 0.3	0.897	0.513
2. Blood loss during pelvic lymph node dissection (ml)	32.5 ± 5.1	35.2 ± 6.5	2.983	0.329
3. Blood loss during prostate excision (ml)	101.6 ± 12.2	123.8 ± 14.1	7.041	0.021
4. Blood loss during bladder-urethral anastomosis (ml)	62.7 ± 9.7	82.5 ± 8.2	8.172	0.019

### Postoperative indicators

Postoperative indicators Postoperative observations for both groups (detailed in Table 3). The number of patients with retained NVB in the 3D group is 21, and in the 2D group, it is 15.The incidence of urinary incontinence in the 3D group was lower than in the 2D group, and the rate of preserved erectile function was higher in the 3D group, with statistically significant differences (*P < *0.05). There were no statistically significant differences between the two groups in terms of postoperative drainage days, hospitalization days, hospitalization costs, time of catheter removal and positive surgical margin rates (*P > *0.05). All 63 specimens were pathologically diagnosed as prostate adenocarcinoma, with the 3D group having Gleason scores of 4–9 and a positive surgical margin rate of 9.1%. The 2D group had Gleason scores of 5–9 and a positive surgical margin rate of 10.0%. All patients were followed up postoperatively for 1–15 months (average 11 months). In the 3D group, there was 1 case of biochemical recurrence and 1 of imaging recurrence; in the 2D group, 2 cases of biochemical recurrence and 1 of imaging recurrence, all satisfactorily controlled with endocrine therapy. No disease-related deaths or other complications were observed in patients from both groups.

**Table 3:** Postoperative indicators.

	3D group	2D group	*t*/*χ^2^*	*p*-value
Postoperative drainage days (d)	3.9 ± 1.3	4.2 ± 1.2	−3.968	0.481
Hospital stay (day)	11.8 ± 1.5	12.5 ± 1.9	2.129	0.528
Hospitalization cost (CNY)	31,326.5 ± 763.5	29,298.3 ± 855.2	4.181	0.247
Urinary incontinence incidence rate	18.2%	30.0%	1.210	0.016
Rate of preserved erectile function	36.4%	26.7%	0.682	0.009
Positive surgical margin rate	9.1%	10.0%	0.519	0.273
Time of catheter removal (day)	6.9 ± 1.1	7.2 ± 1.2	2.001	0.533

## Discussion

For localized prostate cancer (PCa), Surgical approaches mainly include traditional open perineal or retropubic radical prostatectomy (RP), laparoscopic radical prostatectomy (LRP) widely used in recent years, and the latest robot-assisted LRP. Laparoscopic radical prostatectomy can effectively treat early-stage prostate cancer (7), with less trauma compared to open surgery, making it more acceptable to patients (4). However, traditional 2D laparoscopy offers a two-dimensional view, not a three-dimensional one like open surgery. Surgeons need to determine depth perception based on organ positioning and anatomical landmarks, a skill requiring extensive surgical experience and a long learning curve (8). Robot-assisted LRP is an advancement over traditional laparoscopy, consisting of a 3D imaging system, a control console, and a robotic arm surgical system (9, 10). Surgeons can obtain 3D images at the console, controlling simulated arms for precise surgical maneuvers. Compared to traditional laparoscopy, it significantly reduces bleeding, speeds up postoperative recovery, and minimizes damage to the neurovascular bundles around the prostate, better preserving erectile function (11, 12). The major drawback of this system is its high cost and expensive maintenance, potentially increasing the financial burden on patients, thus limiting its widespread adoption. Therefore, the 3D laparoscopic system, also offering a three-dimensional view, has a broad development prospect.

Postoperative urinary incontinence has always been a technical bottleneck in radical prostatectomy. Precise and accurate dissection and protection of the circular muscle group of the bladder neck are crucial for patients to regain urinary control after surgery (13, 14). 3D laparoscopy provides a high-definition three-dimensional view similar to open surgery, clearly displaying the complex pelvic floor structure, allowing the surgeon to operate precisely and maximize the protection of the patient's urinary control function. With the increasing incidence of prostate cancer and a decrease in the age of onset, the preservation of postoperative sexual function is receiving increasing attention from patients. The recovery of erectile function in patients after prostate cancer surgery primarily depends on the preservation of the neurovascular bundle during the operation. The neurovascular bundle, composed of cavernous nerves governing penile erection, runs between the prostate capsule and the levator ani fascia, ascending at the apex of the prostate and finally crossing the urogenital diaphragm. Its anatomical position makes it highly susceptible to injury during LRP, thus necessitating close separation along the prostate capsule during surgery to minimize excessive thermal damage. 3D laparoscopy allows for clear and accurate handling of the aforementioned complex pelvic anatomy, thus maximally protecting the neurovascular bundle and creating favorable conditions for the preservation of postoperative sexual function.

While 3D laparoscopy has its advantages, it also has certain drawbacks: (1) Prolonged surgery or rapid movement of the camera may cause discomfort such as dizziness for the surgeon (15); (2) The camera cannot rotate, unlike 2D laparoscopy, which can obtain different views by rotating the camera angle; (3) When operating in areas like the pelvis, it is prone to interference with other instruments. Additionally, some 3D devices have heavy cameras, which can be physically demanding for assistants who need to hold the laparoscope for extended periods. Laparoscopic radical prostatectomy is complex and time-consuming, with varying degrees of complexity and duration for each step. 3D laparoscopy offers advantages in reducing surgery time and blood loss but also has the aforementioned shortcomings, and its advantages may vary at each step of the surgery. Based on the clinical characteristics of 3D laparoscopy, to better utilize its advantages and avoid its shortcomings, we divided the surgery into four steps: establishing extraperitoneal pneumoperitoneum, pelvic lymph node dissection, prostate excision, and bladder-urethral anastomosis. We studied and compared the advantages and disadvantages of each surgical step under 3D and 2D laparoscopy, switching between 3D and 2D modes based on the specific advantages and disadvantages of each step, selectively using the 3D mode.

Our research found that the 3D group had significantly less operative time and blood loss during the prostate excision and bladder-urethral anastomosis stages compared to the 2D group (*P < *0.05). There was no significant difference in operative time and blood loss during the establishment of extraperitoneal pneumoperitoneum and pelvic lymph node dissection stages (*P *> 0.05). From our surgical experience, (1) There are many blood vessels and nerves controlling urination and sexual function around the prostate, making the anatomy complex. During prostate excision, there's a risk of damaging the urethral sphincter and associated vessels and nerves, leading to complications such as postoperative urinary incontinence. 3D laparoscopy provides an enlarged high-definition three-dimensional view, clearly displaying the anatomical structure and the direction of blood vessels and nerves, allowing for more precise positioning, greatly reducing the difficulty of anatomically separating the prostate, minimizing accidental injuries, and ensuring the patient's urinary control. (2) Bladder-urethral anastomosis has always been a challenging part of radical prostatectomy. Traditional 2D laparoscopy offers a two-dimensional view, making it difficult for surgeons to judge and adjust the angle of needle holding and the direction of needle entry and exit. 3D laparoscopy provides a higher magnification and a high-definition view with depth perception, allowing surgeons to accurately judge the relative position of the instruments to the tissue and adjust the angle and depth of sutures, thereby increasing the speed and quality of suturing and knotting, significantly reducing suture time, and minimizing the risk of needle injury and bleeding. (3) When dealing with easily bleeding areas such as the apex of the prostate, the high-definition three-dimensional view of the 3D laparoscope allows surgeons to clearly identify relatively avascular areas, stay away from the prostate capsule, precisely locate and incise, greatly reducing surgical bleeding. Even in cases of unexpected bleeding during surgery, suturing and electrocoagulation under the 3D view are easier and more precise. (4) Since 3D laparoscopy has a higher magnification than traditional 2D laparoscopy, the camera is positioned further from the target tissue during surgery, reducing the chance of camera contamination. The need for external wiping of the camera lens is significantly reduced, thereby shortening the surgery time to some extent. In summary, Compared to traditional 2D laparoscopy, 3D laparoscopy can shorten the operation time and reduce bleeding in the steps of prostate excision and bladder-urethral anastomosis, but there was no significant difference in peri-operative outcomes.

## Data Availability

The original contributions presented in the study are included in the article/Supplementary Material, further inquiries can be directed to the corresponding authors.

## References

[1] Bray F Ferlay J Soerjomataram I Siegel RL Torre LA Jemal A Global cancer statistics 2018: GLOBOCAN estimates of incidence and mortality worldwide for 36 cancers in 185 countries. CA Cancer J Clin. (2018) 68(6):394–424. 10.3322/caac.21492 30207593

[2] Erratum: global cancer statistics 2018: GLOBOCAN estimates of incidence and mortality worldwide for 36 cancers in 185 countries. CA Cancer J Clin. (2020) 70(4):313. 10.3322/caac.21609 32767693

[3] Makarov DV Humphreys EB Mangold LA Carducci MA Partin AW Eisenberger MA The natural history of men treated with deferred androgen deprivation therapy in whom metastatic prostate cancer developed following radical prostatectomy. J Urol. (2008) 179(1):156–61; discussion 161–2. 10.1016/j.juro.2007.08.133 18001801 PMC4342043

[4] Brown JA Garlitz C Gomella LG McGinnis DE Diamond SM Strup SE Perioperative morbidity of laparoscopic radical prostatectomy compared with open radical retropubic prostatectomy. Urol Oncol. (2004) 22(2):102–6. 10.1016/S1078-1439(03)00101-7 15082005

[5] Tanagho YS Andriole GL Paradis AG Madison KM Sandhu GS Varela JE 2D versus 3D visualization: impact on laparoscopic proficiency using the fundamentals of laparoscopic surgery skill set. J Laparoendosc Adv Surg Tech A. (2012) 22(9):865–70. 10.1089/lap.2012.0220 23072406

[6] Davenport K Burns A Helo S Bailey G Peters C Schenkman N. 1510 comparison of 3d stereoscope vs standard 2d laparoscope for performance of two standard laparoscopic tasks by urology residents. J Urol. (2012) 187(4):611. 10.1016/j.juro.2012.02.1277

[7] Schroeck FR Krupski TL Sun L Price MM Polascik TJ Robertson CN Satisfaction and regret after open retropubic or robot-assisted laparoscopic radical prostatectomy. Eur Urol. (2008) 54(4):785–93. 10.1016/j.eururo.2008.06.063 18585849

[8] Patel HR Ribal MJ Arya M Nauth-Misir R Joseph JV. Is it worth revisiting laparoscopic three-dimensional visualization? A validated assessment. Urology. (2007) 70(1):47–9. 10.1016/j.urology.2007.03.014 17656206

[9] Sood A Grauer R Jeong W Butaney M Mukkamala A Borchert A Evaluating post radical prostatectomy mechanisms of early continence. Prostate. (2022) 82(12):1186–95. 10.1002/pros.24371 35579026

[10] Deng W Zhang C Jiang H Li Y Zhu K Liu X Transvesical versus posterior approach to retzius-sparing robot-assisted radical prostatectomy: a retrospective comparison with a 12-month follow-up. Front Oncol. (2021) 11:641887. 10.3389/fonc.2021.641887 33937043 PMC8082308

[11] Hamdy FC Donovan JL Lane JA Mason M Metcalfe C Holding P 10-year outcomes after monitoring, surgery, or radiotherapy for localized prostate cancer. N Engl J Med. (2016) 375(15):1415–24. 10.1056/NEJMoa1606220 27626136

[12] Wilt TJ Jones KM Barry MJ Andriole GL Culkin D Wheeler T Follow-up of prostatectomy versus observation for early prostate cancer. N Engl J Med. (2017) 377(2):132–42. 10.1056/NEJMoa1615869 28700844

[13] Hagman A Lantz A Carlsson S Höijer J Stranne J Tyritzis SI Urinary continence recovery and oncological outcomes after surgery for prostate cancer analysed by risk category: results from the LAParoscopic prostatectomy robot and open trial. World J Urol. (2021) 39(9):3239–49. 10.1007/s00345-021-03662-0 33743059

[14] Ploussard G. Robotic surgery in urology: facts and reality. What are the real advantages of robotic approaches for prostate cancer patients? Curr Opin Urol. (2018) 28(2):153–8. 10.1097/MOU.0000000000000470 29232271

[15] Zhang L Zhang YQ Zhang JS Xu L Jonas JB. Visual fatigue and discomfort after stereoscopic display viewing. Acta Ophthalmol. (2013) 91(2):149–53. 10.1111/aos.12006 23164154

